# Multi-optimization for thermal deformation of gravitational wave telescope based on CFRP characteristics

**DOI:** 10.1038/s41598-024-65836-1

**Published:** 2024-06-28

**Authors:** Bohong Li, Yong Yan, Jian Luo, Sijun Fang, Rui Zhang, Hongwen Hai, Yehao Cao

**Affiliations:** MOE Key Laboratory of TianQin Mission, TianQin Research Center for Gravitational Physics & School of Physics and Astronomy, Frontiers Science Center for TianQin, Gravitational Wave Research Center of CNSA, Sun Yat-Sen University (Zhuhai Campus), Zhuhai, 519082 China

**Keywords:** Telescope thermal deformation, Unconventional CFRP layup design, CTE optimization, NSGA-II algorithm, Astronomy and planetary science, Engineering, Materials science, Structural materials

## Abstract

Gravitational wave telescope place extremely high demands on structural thermal deformation, making material selection a critical issue. Carbon fiber reinforced polymer (CFRP) is an ideal choice for the support structure of telescope due to its low coefficient of thermal expansion (CTE) and designable properties. However, current research on the optimization of the CTE of CFRP is scarce, and conventional methods struggle to find layups that meet the requirements. In this paper, an unconventional layup optimization method is proposed to solve this problem. Initially defining the characteristics of the telescope structure and using different layup material for the main and side support rods to minimize thermal deformation. Subsequently, the NSGA-II algorithm is used to optimize the layups which are divided into conventional and unconventional layups. Specimens are then produced from these results and tested to assess the impact of processing errors on practical applications. The results demonstrate that the optimized CFRP meet the CTE requirements and, when applied to the structure, significantly reduces the thermal deformation in the eccentric direction compared to conventional designs. Additionally, a numerical analysis evaluates the effect of ply orientation errors on the performance of unconventional layups, discussing the method's limitations within these contexts.

## Introduction

TianQin (TQ) is a project to detect gravitational wave in the frequency band from 0.1 mHz to 1 Hz. It plans to launch three identical satellites into deep space orbits and use optical interferometry technology to precisely measure the distances between them in order to detect the tiny changes in distance caused by gravitational waves ^1^. The telescope plays a crucial role as a key component of the satellite's core payload. Any slight structural deformation can affect the optical signal, which directly impacts the accuracy of the measurement results^2^. Therefore, a stable structure design is necessary to minimize the thermal deformation of the telescope structure.

The optical sensitivity analysis indicates that the axial change rate of the secondary mirror must be less than 0.55 μm/K and the eccentric rate of change must be less than 0.2 μm/K. Consequently, the structure's overall thermal deformation coefficient must be below 0.6 × 10^–6^/K. Common aerospace materials that meet this requirement include Zerodur, Invar alloy, and Carbon fiber reinforced polymer (CFRP). The coefficient of thermal expansion (CTE) of Zerodur, a kind of extremely low thermal expansion glass, can be as low as 10^–8^/K or lower, making it a popular choice for manufacturing optical components in aerospace systems^3,4^. However, it is difficult to achieve the desired design and assembly of the structure due to its brittle nature and low toughness and strength. Invar alloy is a customizable material that can be engineered to reduce its CTE to less than 10^–8^/K. Compared to Zerodur, it has good mechanical properties and is easy to machine and assemble structures. However, its high density increases the weight of spacecraft components, and as a magnetic material, Invar could also interfere with other internal components ^5^. CFRP is a designable material with high stiffness. Its CTE can be customized to meet specific requirements through layup design, making it the ideal choice for the current telescope prototype structure^6,7^. Therefore, accurately and efficiently optimizing the CFRP layup to meet the precise requirements of gravitational wave detection is a key issue.

Previous studies on CFRP layup optimization and application have primarily focused on lightweight design^8,9^ and optimization of mechanical properties^10–13^. These studies typically limited the orientation of the laminae to conventional angles, such as ± 45°, 0°, and 90°, which are known to enhance mechanical design efficiency and achieve superior mechanical properties in longitudinal, transverse, and shear directions^14–17^. Nevertheless, given that the main challenge for the TQ telescope at this stage is thermal deformation of the structure, with lesser emphasis on mechanical properties, unconventional angle layups become a viable option. In contrast, conventional angle layups offer fewer options. Therefore, given that the mechanical properties of the telescope meet the requirements, determining how to use unconventional angle layups to reduce thermal deformation has become a critical issue.

Some studies have used unconventional angle layup designs to enhance the performance of CFRP. The Three-mirror Anastigmat (TMA) telescope designed by Lin et al. used unconventional angle laminations^18^. Similarly, Vermes et al. optimized and analyzed the lightweight and failure issues of unconventional CFRP laminates^19^. Furthermore, Pathak et al. conducted research on the mechanical and thermal properties and destructibility of CFRP, concluding that fiber orientation significantly influences these properties^20^. Most of these studies focus on mechanical properties, such as the balance between thickness and lightweight, failure buckling. Research and application of CTE in CFRP are limited. Sun et al. demonstrated laminates with very low CTE at unconventional angles during characterization tests^21^. Experiments comparing ultra-low expansion CFRP with Zerodur have shown the significant potential of unconventional layups to reduce structural thermal deformation. However, in many studies, a specific layer is often selected as the variable θ, and an exhaustive method is then employed to evaluate the design^22–24^. While this method can identify a CFRP laminate that meets the requirements, it is inefficient and might not yield the optimal layup. Therefore, there is a need for a universal optimization method for CFRP thermal deformation, which could solve the laminates design problems more efficiently when applying CFRP structures broadly.

Since CFRP material is a laminated structure with anisotropic characteristics, its optimization involves multi-objective and multi-variable problems. Extensive research has been conducted on optimizing CFRP layup methods, involving machine learning, neural networks, finite element methods, and experimental approaches^25–30^. Among the various approaches explored, the Non-dominated Sorting Genetic Algorithm II (NSGA-II) stands out as particularly suitable for the challenges addressed in this study. NSGA-II is a classical genetic algorithm for multi-objective optimization. It is known for its fast execution speed and good convergence of solution sets and is often used as a benchmark for the performance of other algorithms^31^. Previous research has shown the potential of the NSGA-II algorithm for CFRP layup optimization^32^. Sun et al. used the NSGA-II algorithm to study thin-wall structures under crash conditions^33^. Zhang et al. conducted a comprehensive optimization of bistable laminates using the NSGA-II algorithm, and the results demonstrate a notable enhancement in the performance of the optimized structure^34^. Aydin and Artem first proposed using the NSGA-II algorithm to optimize the CTE of CFRP^35^, but practical applications of this specific use are still limited^36^. For gravitational wave detection telescopes, Laser Interferometer Space Antenna (LISA) has previously studied the CTE of CFRP materials^37–39^, and has given a prototype of a truss telescope made using CFRP materialsy^40^. Among the three main thermal deformation degrees of freedom in the telescope, the axial deformations (dz) of the primary and secondary mirrors can almost meet the requirements. The tilting degree of freedom (dRx) requires analysis in conjunction with the mirror frame configuration; therefore, this paper does not delve into detailed discussion. It is important to highlight that the eccentricity degree of freedom (dy) exhibits poor thermal deformation performance, which is an order of magnitude greater than the design specifications. Thus, a crucial aspect of applying CFRP is optimizing the structural thermal deformation (dy) related to the telescope's eccentricity, to ensure that the design specifications are met.

Building on previous research, this study begins with the objective optimization of CFRP, delivering application simulations that integrate layup design with the structural requirements of the telescope. In Section "Determination of optimized design indicators", the thermal deformation model of the telescope truss structure is established, and the design objectives for the materials are defined. Section "Mathematical modeling of thermal deformation of CFRP" discusses the thermal deformation theory of composite materials, and Section "Optimizing CFRP layup by NSGA-II" introduces the optimization application method by integrating it with the NSGA-II algorithm. Sections "Optimization design results" and "Experimental results and error analysis" present the optimization results and experimental validation of the CFRP layups. Section "Application in gravitational wave telescope design" integrates the optimized CFRP material into the structure and verifies the validity of the CFRP optimization results through finite element simulation. Section “Discussion of limitations of optimization methods” discusses the limitations of the current process and optimization method. The conclusions of the study are presented in section "Conclusion".

## Methods and optimization

### Determination of optimized design indicators

**Figure 1:**
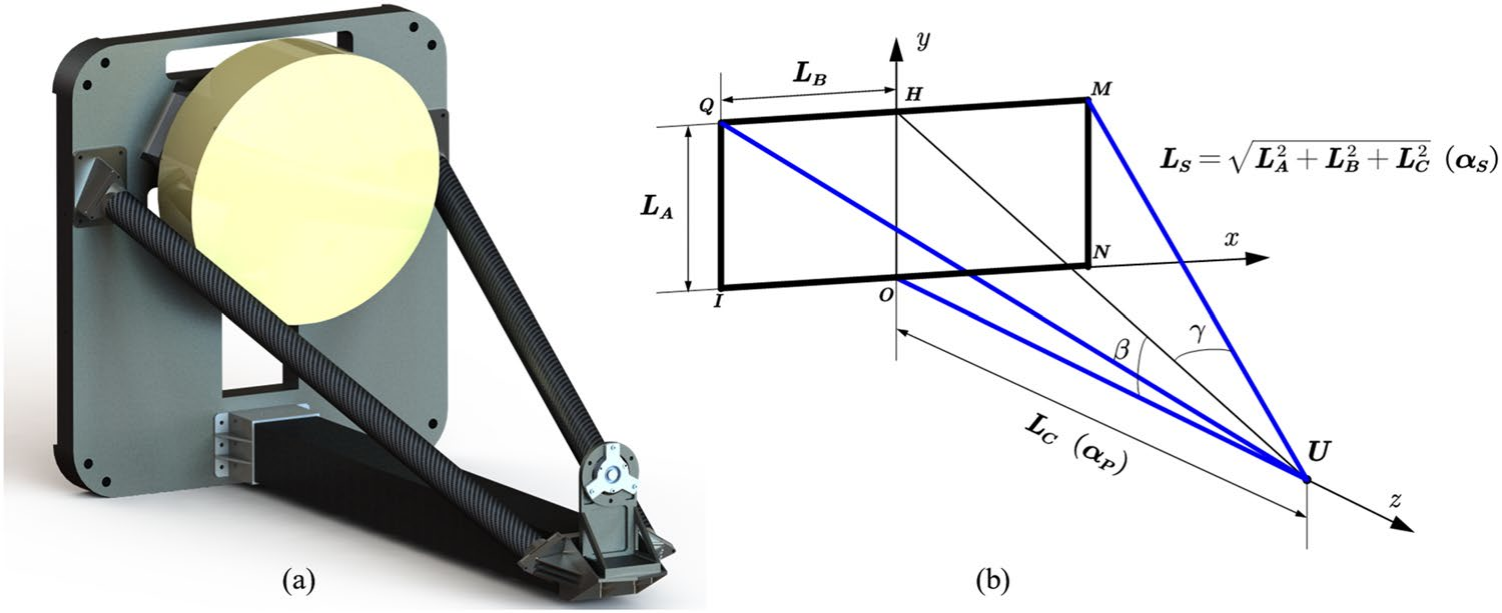
Solid Model (**a**); Theoretical Model (**b**).

To investigate the relationship between truss parameters and thermal deformation, the solid model of the telescope support structure has been simplified to a truss model. As illustrated in Fig. 1, QU and MU denote the two side support rods, while OU represents the bottom main support rod, which is perpendicular to the xOy plane. Additionally, ${\alpha }_{P}$ and ${\alpha }_{S}$ refer to the CTE for the main and side rods, respectively, as shown in Fig. 1b.

**Figure 2:**
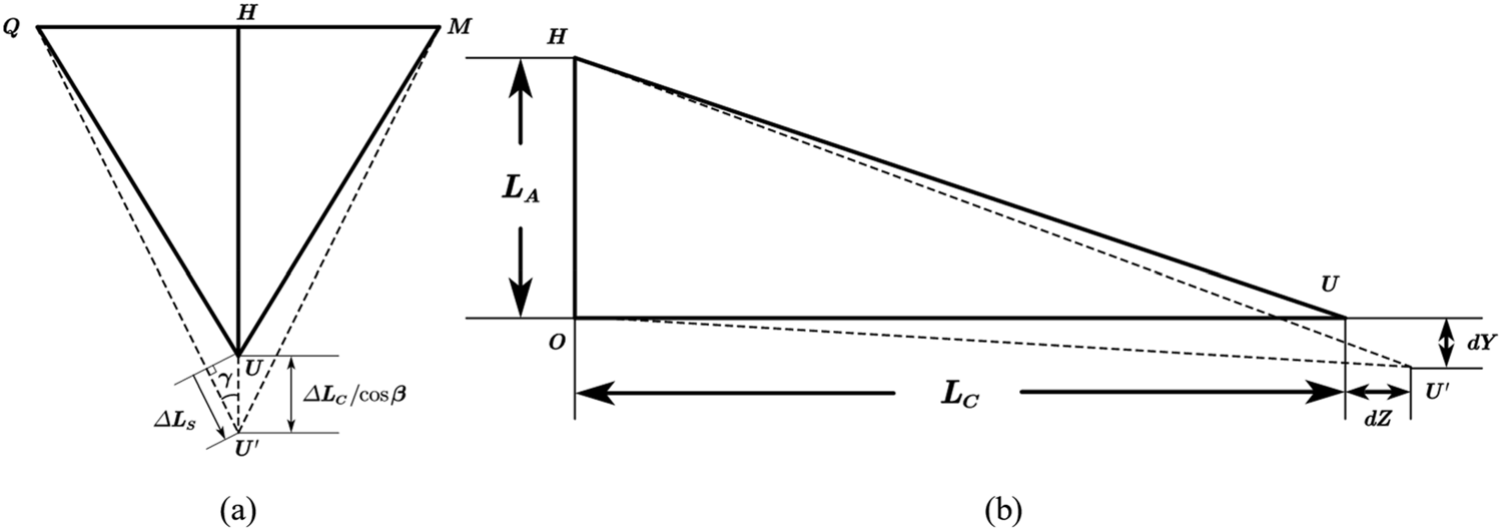
Projections of the truss model on the QMU (**a**) and YOZ (**b**) planes.

Truss theory posits that truss rods undergo only axial deformation, without experiencing bending, shearing, or other forms of deformation, as illustrated in Fig. 2. The projection onto the yOz plane demonstrates that the secondary mirror's point U moves to point U' following deformation. The relative deformations, dz and dy, represent the thermal deformation of the secondary mirror's position within the truss support structure.

Considering the deformation coordination relationship within the truss:

1 $$ \begin{array}{*{20}c} {\begin{array}{*{20}c} {\Delta L_{S} = \frac{{\Delta L_{C} }}{\cos \beta } \times \cos \gamma } \\ \end{array} } \\ \end{array} $$

There are geometric relationships:

2 $$ \begin{array}{*{20}c} {\begin{array}{*{20}c} {\left\{ {\begin{array}{*{20}l} {L_{S} = \sqrt {L_{A}^{2} + L_{B}^{2} + L_{C}^{2} } } \hfill \\ {\cos \beta = L_{C} /\sqrt {L_{A}^{2} + L_{C}^{2} } } \hfill \\ {\cos \gamma = \sqrt {L_{A}^{2} + L_{C}^{2} } /\sqrt {L_{A}^{2} + L_{B}^{2} + L_{C}^{2} } } \hfill \\ \end{array} } \right.} \\ \end{array} } \\ \end{array} $$

The basic equation for thermal expansion is $\Delta L = L \times \alpha \times \Delta T$, which is inserted into the Eqs. (1) and (2) to obtain the thermal deformation of the node U as a function of the geometric parameters of the structure:

3 $$ \begin{array}{*{20}c} {dy = \frac{{L_{A}^{2} + \left( {L_{C} + \alpha_{P} L_{C} \Delta T} \right)^{2} - \left( {L_{S} \times \cos \gamma + \frac{{\alpha_{S} L_{S} \Delta T}}{\cos \gamma }} \right)^{2} }}{{2L_{A} }}} \\ \end{array} $$

4 $$ \begin{array}{*{20}c} {dz = \sqrt {\left( {L_{C} + L_{C} \alpha_{P} \Delta t} \right)^{2} - \left( {dy} \right)^{2} } - L_{C} } \\ \end{array} $$

**Figure 3:**
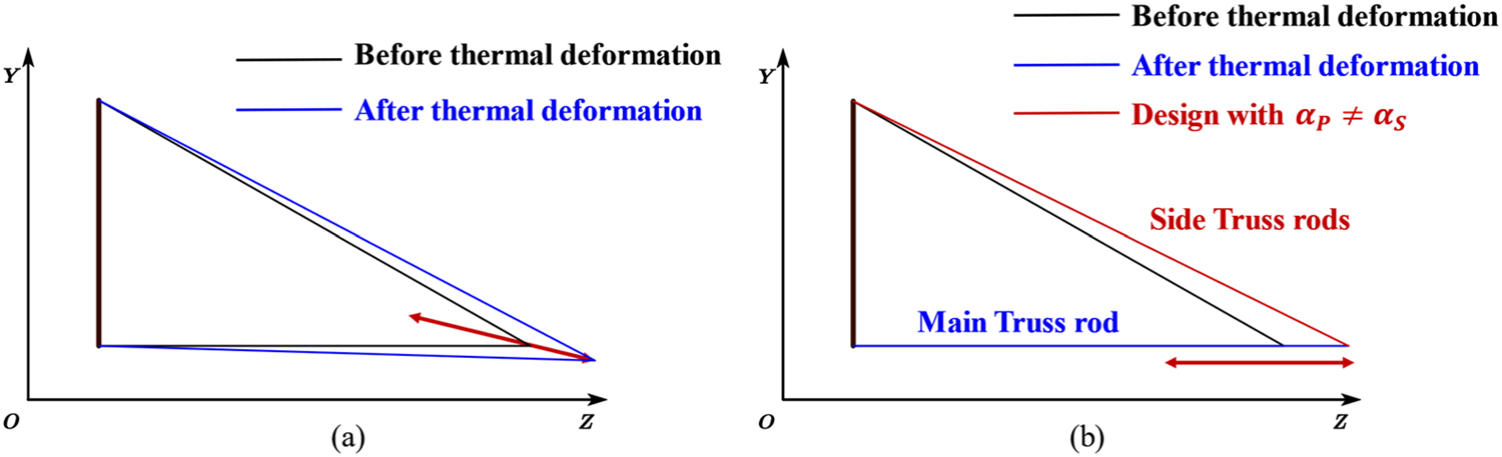
The schematic diagram of differentiated structure design principles: ${\alpha }_{S}={\alpha }_{P}$(**a**) and ${\alpha }_{S}\ne {\alpha }_{P}$(**b**).

In the general design of truss structures, the same material is typically chosen for all support rods, denoted as $\alpha_{{\text{s}}} = \alpha_{{\text{P}}}$. However, it has been found that the structural thermal deformation, dy, can be reduced by assigning different values such that $\alpha_{{\text{S}}} \ne \alpha_{{\text{P}}}$, as demonstrated by Eq. (3). The principle of the optimized design is shown in Fig. 3.

Based on the current design of the telescope prototype, the basic structural design parameters are as follows: *L*_A_ = 326 mm, *L*_B_ = 215 mm, *L*_C_ = 838 mm, and the global space temperature change of the telescope during the orbital phase is ΔT = 1 K.

The objective of the structural design is to minimize the thermal deformation of the structure, and the observation of Eq. (3) reveals that changing the thermal deformation dy in the direction of deviation is achievable with different parameter pairings to achieve dy = 0. Therefore, the parameters with dy = 0 are inserted into Eq. (3) to obtain the short form:

5 $$ \begin{array}{*{20}c} {\begin{array}{*{20}c} {\frac{{5\left( {170949\alpha_{S} + 161704} \right)^{2} }}{105431008} = \frac{{175561\left( {\alpha_{P} + 1} \right)^{2} }}{163} + 163} \\ \end{array} } \\ \end{array} $$

This indicates that when $\alpha_{s}$ and $\alpha_{P}$ satisfy the numerical relationship defined in Eq. (5), the structure undergoes no thermal deformation in the direction of eccentricity. At this point, the ratio of ${\alpha }_{P}$ to ${\alpha }_{S}$ is 1.217:1. According to Eqs. (3), (4), and (5), to optimize the thermal deformation of the structure, three design indicators must be identified:

1. The first design indicator is to ensure dy = 0, the axial thermal expansion coefficient ratio of the main and side support rod materials should be 1.217:1.
2. When dy = 0, the axial thermal deformation of the structure is $dz={L}_{C}{\alpha }_{P}\Delta t$. The optical system requires that under conditions of a unit temperature change, dy < 200 nm/K and dz < 550 nm/K. Therefore, the CTE of the main support rod material should be $\alpha_{P} < 0.6 \times 10^{ - 6} /{\text{K}}$.
3. The above truss structures $\alpha_{P} , \alpha_{S}$ both represent the axial thermal expansion coefficient of the support rods. However, CFRP is characterized by anisotropy, leading to different CTEs in the axial and radial directions of the rods. To avoid thermal stress mismatch between CFRP rods and metal connections, the radial thermal deformation coefficient of the material is also considered. Thus, the conventional wisdom that smaller is better when it comes to CTE is not necessarily applicable in this case.

**Figure 4:**
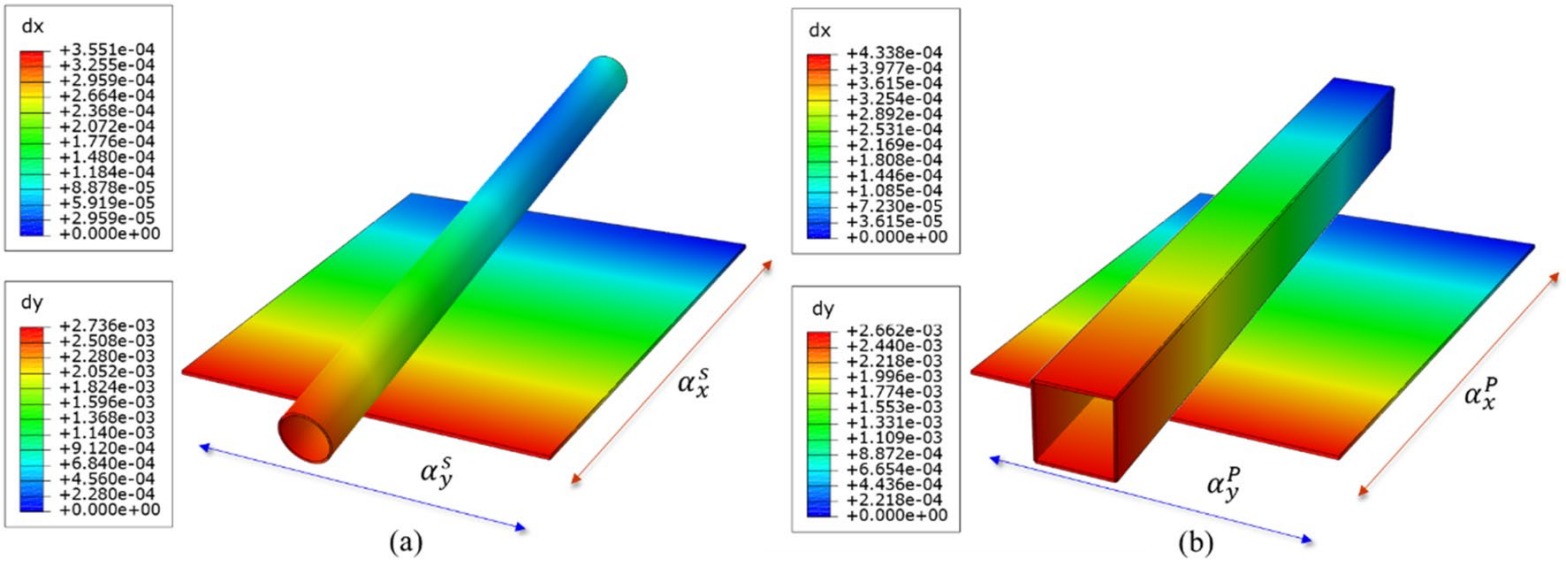
Schematic diagram of CFRP optimization objectives: ${\alpha }_{x}^{S},{\alpha }_{y}^{S},{\alpha }_{x}^{P},{\alpha }_{y}^{P}$ with side rods (**a**); main rod (**b**).

The laminates' in-plane x-direction CTE, representing the axial thermal expansion of the rods, is denoted by ${\alpha }_{x}$, while the radial thermal expansion is represented by ${\alpha }_{y}$. Notably, the joints in the structure are all made of Invar, with CTE of ${\alpha }_{Invar}=1\times {10}^{-6}/K$. Thus, a third design indicator emerges: it is crucial not only to ensure that ${\alpha }_{x}$ in each support rod meets the specified requirements, but also to adjust ${\alpha }_{y}$ to be as close as possible to $1\times {10}^{-6}/K$. Figure 4 illustrates the orientation correspondence between the support rods and the material. The simulation in this figure uses millimeter units to analyze the thermal expansion of CFRP laminates and support rods per unit length (L = 1 m) under conditions of unit temperature ($\Delta {\text{T}} = 1\;{\text{K}}$) change.

In summary, given the need to perform layup optimization design separately for the main and side rods, there are four optimization objectives: ${\alpha }_{x}^{S},{\alpha }_{y}^{S},{\alpha }_{x}^{P},{\alpha }_{y}^{P}$. Thus, there are three requirements for the CTE of materials: 1) ${\alpha }_{x}^{S}$ : ${\alpha }_{x}^{P}$ = 1:1.217; 2) ${\alpha }_{x}^{P}<0.6\times {10}^{-6}/K$; 3) ${\alpha }_{y}^{P}, {\alpha }_{y}^{S}$ as close to $1\times {10}^{-6}/K$.

### Mathematical modeling of thermal deformation of CFRP

From the above analysis, it is clear that the core of the thermal deformation of the structure lies in the CTE of the three truss rods. Since this value can be altered by designing the angle of the CFRP material layup, it is necessary to establish a functional relationship between the CTE of laminates and the orientation of the lamina.

**Figure 5:**
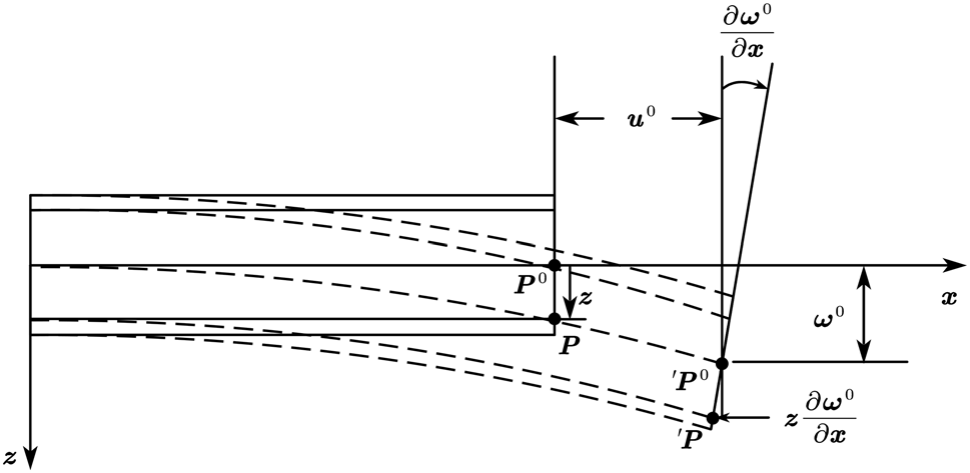
Schematic diagram of the deformation of a laminated composite material structure as a beam structure.

CFRP is obtained from a single layer of plate after lamination. The laminates can be considered as a beam structure with continuous variation in the thickness direction, as shown in Fig. 5.

Based on the diagram, it can be known:

6 $$ \begin{array}{*{20}c} {\left[ {\begin{array}{*{20}c} {\varepsilon_{x}^{0} } \\ {\varepsilon_{y}^{0} } \\ {\gamma_{xy}^{0} } \\ \end{array} } \right] = \left[ {\begin{array}{*{20}c} {\frac{{\partial u_{0} }}{\partial x}} \\ {\frac{{\partial v_{0} }}{\partial y}} \\ {\frac{{\partial u_{0} }}{\partial y} + \frac{{\partial v_{0} }}{\partial x}} \\ \end{array} } \right],\left[ {\begin{array}{*{20}c} {{\rm K}_{x} } \\ {{\rm K}_{y} } \\ {{\rm K}_{xy} } \\ \end{array} } \right] = \left[ {\begin{array}{*{20}c} { - \frac{{\partial^{2} \omega_{0} }}{{\partial x^{2} }}} \\ { - \frac{{\partial^{2} \omega_{0} }}{{\partial y^{2} }}} \\ { - 2\frac{{\partial^{2} \omega_{0} }}{\partial x\partial y}} \\ \end{array} } \right]} \\ \end{array} $$

Strain formula can be obtained:

7 $$ \left[ {\begin{array}{*{20}c} {\varepsilon_{x} } \\ {\varepsilon_{y} } \\ {\gamma_{xy} } \\ \end{array} } \right] = \left[ {\begin{array}{*{20}c} {\varepsilon_{x}^{0} } \\ {\varepsilon_{y}^{0} } \\ {\gamma_{xy}^{0} } \\ \end{array} } \right] + z\left[ {\begin{array}{*{20}c} {{\rm K}_{x} } \\ {{\rm K}_{y} } \\ {{\rm K}_{xy} } \\ \end{array} } \right] $$

From the structural strain, the structural stress of each layer can be deduced in reverse, ${Q}_{k}$ is the off-axis stiffness matrix for each layer:

8 $$ \begin{array}{*{20}c} {\left[ {\begin{array}{*{20}c} {\sigma_{x} } \\ {\sigma_{y} } \\ {\tau_{xy} } \\ \end{array} } \right]_{k} = \left[ {\begin{array}{*{20}c} {Q_{11} } & {Q_{12} } & {Q_{16} } \\ {Q_{12} } & {Q_{22} } & {Q_{26} } \\ {Q_{16} } & {Q_{26} } & {Q_{66} } \\ \end{array} } \right]_{k} \left[ {\begin{array}{*{20}c} {\varepsilon_{x} } \\ {\varepsilon_{y} } \\ {\gamma_{xy} } \\ \end{array} } \right]_{k} } \\ \end{array} $$

By integrating the stress of each layer in the thickness direction, the following can be obtained:

9 $$ \begin{array}{*{20}c} {\left\{ {\begin{array}{*{20}l} {N_{x} = \mathop \smallint \limits_{{ - \frac{h}{2}}}^{\frac{h}{2}} \sigma_{x} dz = \mathop \smallint \limits_{{ - \frac{h}{2}}}^{\frac{h}{2}} \left[ {Q]_{kx} } \right[\varepsilon ]_{kx} dz} \hfill \\ {M_{x} = \mathop \smallint \limits_{{ - \frac{h}{2}}}^{\frac{h}{2}} \sigma_{x} zdz = \mathop \smallint \limits_{{ - \frac{h}{2}}}^{\frac{h}{2}} \left[ Q \right]_{kx} \left[ \varepsilon \right]_{kx} zdz} \hfill \\ \end{array} } \right.} \\ \end{array} $$

where $N_{x}$ and $M_{x}$ are the resultant force and moment per unit length on the laminated composite material in the x direction of the plane. The same applies to the y direction and the shear direction x–y.

Obtaining the constitutive relationship of laminated composite materials:

10 $$\begin{array}{c}\left[\begin{array}{c}\begin{array}{c}{N}_{x}\\ {N}_{y}\\ {N}_{xy}\end{array}\\ {M}_{x}\\ {M}_{y}\\ {M}_{xy}\end{array}\right]=\left[\begin{array}{cc}\begin{array}{ccc}{A}_{11}& {A}_{12}& {A}_{16}\\ {A}_{12}& {A}_{22}& {A}_{26}\\ {A}_{16}& {A}_{26}& {A}_{66}\end{array}& \begin{array}{ccc}{B}_{11}& {B}_{12}& {B}_{16}\\ {B}_{12}& {B}_{22}& {B}_{26}\\ {B}_{16}& {B}_{26}& {B}_{66}\end{array}\\ \begin{array}{ccc}{B}_{11}& {B}_{12}& {B}_{16}\\ {B}_{12}& {B}_{22}& {B}_{26}\\ {B}_{16}& {B}_{26}& {B}_{66}\end{array}& \begin{array}{ccc}{D}_{11}& {D}_{12}& {D}_{16}\\ {D}_{12}& {D}_{22}& {D}_{26}\\ {D}_{16}& {D}_{26}& {D}_{66}\end{array}\end{array}\right]\left[\begin{array}{c}\begin{array}{c}{\varepsilon }_{x}^{0}\\ {\varepsilon }_{y}^{0}\\ {\gamma }_{xy}^{0}\end{array}\\ {K}_{x}\\ {K}_{y}\\ {K}_{xy}\end{array}\right]\end{array}$$

${A}_{ij}$ is the stiffness coefficient that relates the resultant force to the mid-surface strain; ${D}_{ij}$ and ${B}_{ij}$ indicate the coupling of the orthogonal deformation to other directions.

The objective of this work is to optimize for the thermal deformation of the material. The reason is that the thermal expansion does not involve bending stresses and the order of stacking of constraint layers is symmetrical, which prevents the coupled deformation of the material tension-bending-shear, so that ${B}_{ij}={D}_{ij}=0$. Therefore, Eq. (10) is simplified and inverted:

11 $$ \begin{array}{*{20}c} {\left[ {\begin{array}{*{20}c} {\varepsilon_{x}^{0} } \\ {\varepsilon_{y}^{0} } \\ {\gamma_{xy}^{0} } \\ \end{array} } \right] = \left[ {\begin{array}{*{20}c} {A_{11}^{\prime } } & {A_{12}^{\prime } } & {A_{16}^{\prime } } \\ {A_{12}^{\prime } } & {A_{22}^{\prime } } & {A_{26}^{\prime } } \\ {A_{16}^{\prime } } & {A_{26}^{\prime } } & {A_{66}^{\prime } } \\ \end{array} } \right]\left[ {\begin{array}{*{20}c} {N_{x}^{T} } \\ {N_{y}^{T} } \\ {N_{xy}^{T} } \\ \end{array} } \right]} \\ \end{array} $$

${A}_{ij}^{\prime}$ indicates that the element is from the inverse matrix of the original tensile stiffness matrix $\left[A\right]$; $\left[{N}^{T}\right]$ represents the thermal stress per unit width experienced. Since the model of the thermal expansion of the material does not take external forces into account, the stress of each layer is $[{\sigma ]}_{k}=\Delta T{\left[Q\right]}_{k}[{\alpha ]}_{k}$.The relationship between the $\left[{N}^{T}\right]$ and CTE of each layer is determined as follows:

12 $$ \begin{array}{*{20}c} {\left[ {N^{T} } \right] = \left[ {\begin{array}{*{20}c} {N_{x}^{T} } \\ {N_{y}^{T} } \\ {N_{xy}^{T} } \\ \end{array} } \right] = \Delta T\mathop \sum \limits_{k = 1}^{n} \left[ Q \right]_{k} \left[ {\begin{array}{*{20}c} {\alpha_{x} } \\ {\alpha_{y} } \\ {\alpha_{xy} } \\ \end{array} } \right]_{k} \left( {z_{k} - z_{k - 1} } \right)} \\ \end{array} $$

Meanwhile, the relationship between the elements in the tensile stiffness matrix $[A]$ and the stiffness matrix of each layer is:

13 $$ \begin{array}{*{20}c} {A_{ij} = \mathop \sum \limits_{k = 1}^{n} \left( {Q_{ij} } \right)_{k} \left( {z_{k} - z_{k - 1} } \right)} \\ \end{array} $$

The material strain vector $[\varepsilon ]$ for a certain temperature difference $\Delta T$ is obtained by substituting $[{N}^{T}]$ and ${A}_{ij}$ in Eq. (11), and the relationship between the coefficient of thermal expansion of the laminate ${\left[\alpha \right]}_{l}$ and the orientation of the lamina is obtained by integration:

14 $$ \begin{array}{*{20}c} {\left[ \alpha \right]_{l} = \left[ A \right]^{ - 1} \mathop \sum \limits_{k = 1}^{n} \left[ t \right]_{k}^{ - 1} \left( {z_{k} - z_{k - 1} } \right)\left( {\left[ Q \right]_{k} \left[ \alpha \right]_{k} } \right)} \\ \end{array} $$

$\left[ t \right]$ is the coordinate transformation matrix. Due to the symmetry of the layup, thermal expansion occurs only in the orthogonal directions, and the coupling thermal expansion coefficient ${\alpha }_{xy}=0$. Therefore, Eq. (14) shows that ${\alpha }_{x}$ and ${\alpha }_{y}$ within the laminate are adjusted by the orientation of the lamina.

### Optimizing CFRP layup by NSGA-II

**Figure 6:**
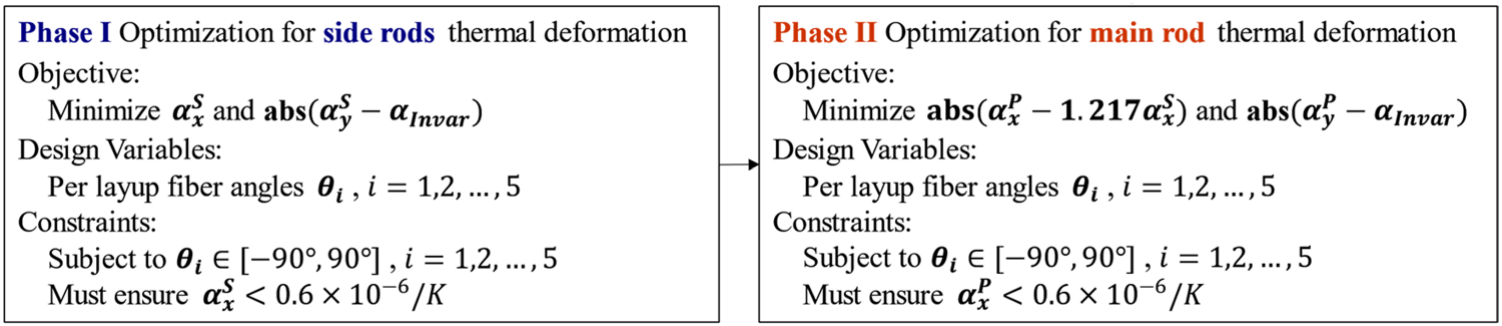
Layer optimization process.

This paper performs layup optimization in two stages: first, considering the lower CTE of the side support rods than the main rod, the layup for the side support rods is optimized to achieve the minimum ${\alpha }_{x}^{S},{\alpha }_{y}^{S}$; then, the layup of the main support rod is optimized in a ratio of 1:1.217 to obtain ${\alpha }_{x}^{P},{\alpha }_{y}^{P}$, as shown in Fig. 6.

To meet the requirements for structural strength and geometric dimensions, 20 layers of 0.15 mm thick M40JB/C1414 CFRP are used. Table 1 shows the performance of the alternative materials for the telescope structure design. The Zerodur was supplied by SCHOTT with the specification of ZERODUR® Expansion Class 1. Invar alloys was supplied by Beijing Aerospace Invar Materials Technology Development Corporation, which is a kind of modified alloy based on 4J32. CFRP was supplied by Chang-Chun Long Aerospace Composite materials Co., Ltd. with the fiber specification M40J, and the matrix material was selected as C1414 epoxy resin.

**Table 1:** Candidate materials for truss support structure of telescope prototype.

Material	Specification	Density	Engineering constants	CTE
Zerodur	ZERODUR® Expansion Class 1	$\rho = 2530\;{\text{kg}}/{\text{m}}^{3}$	E = 91GPa, *v* = 0.24	$\alpha = 0.05 \times 10^{ - 6} /{\text{K}}$
Invar	4J32/B	$\rho = 7980\;{\text{kg/m}}^{{3}}$	E = 168GPa, *v* = 0.28	$\alpha = 1 \times 10^{ - 6} /{\text{K}}$
CFRP	Carbon Fiber: M40J Epoxy resin matrix: C1414	$\rho = 1980\;{\text{kg/m}}^{{3}}$	E1 = 188.0Gpa, *v*_12_ = 0.312 E2 = 7.17Gpa G12 = 4.3GPa G23 = 2.8GPa	$\alpha_{1} = - 0.225 \times 10^{ - 6} /{\text{K}}$ $\alpha_{2} = 35.102 \times 10^{ - 6} /{\text{K}}$

To prevent twisting and bending coupling effects, the laminate should be laid in a complementary and symmetrical manner. The first 10 layers and the last 10 layers must be symmetric. Within these 10 layers, it is necessary to ensure the presence of layups with identical positive and negative angles. This arrangement requires optimization for five ply angles, referred to as $\left( { \pm \theta_{1} , \pm \theta_{2} , \pm \theta_{3} , \pm \theta_{4} , \pm \theta_{5} } \right)_{s}$, where $\theta_{i} \in \left[ { - 90^{^\circ } ,90^{^\circ } } \right]$. Normally, the optimization of layup design involves directly listing several common layups and selecting the one that is closest to the design goal as the design scheme. This method is simple and convenient but lacks precision. Therefore, this paper presents optimization of conventional and unconventional ply angles. Conventional ply angles are fixed at [± 45°, ± 30°, ± 60°, 0°, 90°] and can be controlled by variable constraints in the optimization program. Unconventional layups are not constrained during optimization.

The study utilizes the NSGA-II algorithm, which requires conflicts between different optimization objectives to find a set of design solutions forming a 'Pareto front'. The results on the Pareto front represent extreme states, where improving one objective function often implies a regression in another, ensuring that all optimization results are relatively optimal solutions. For laminate design, ${\alpha }_{x}$ and ${\alpha }_{y}$ are conflicting objective functions, making the NSGA-II algorithm particularly suitable for optimizing the CTE issue of CFRP.

Moreover, the advancement of NSGA-II lies in its introduction of the concepts of 'crowding distance' and 'non-dominated sorting', ensuring the effectiveness and diversity of the optimization results.

**Figure 7:**
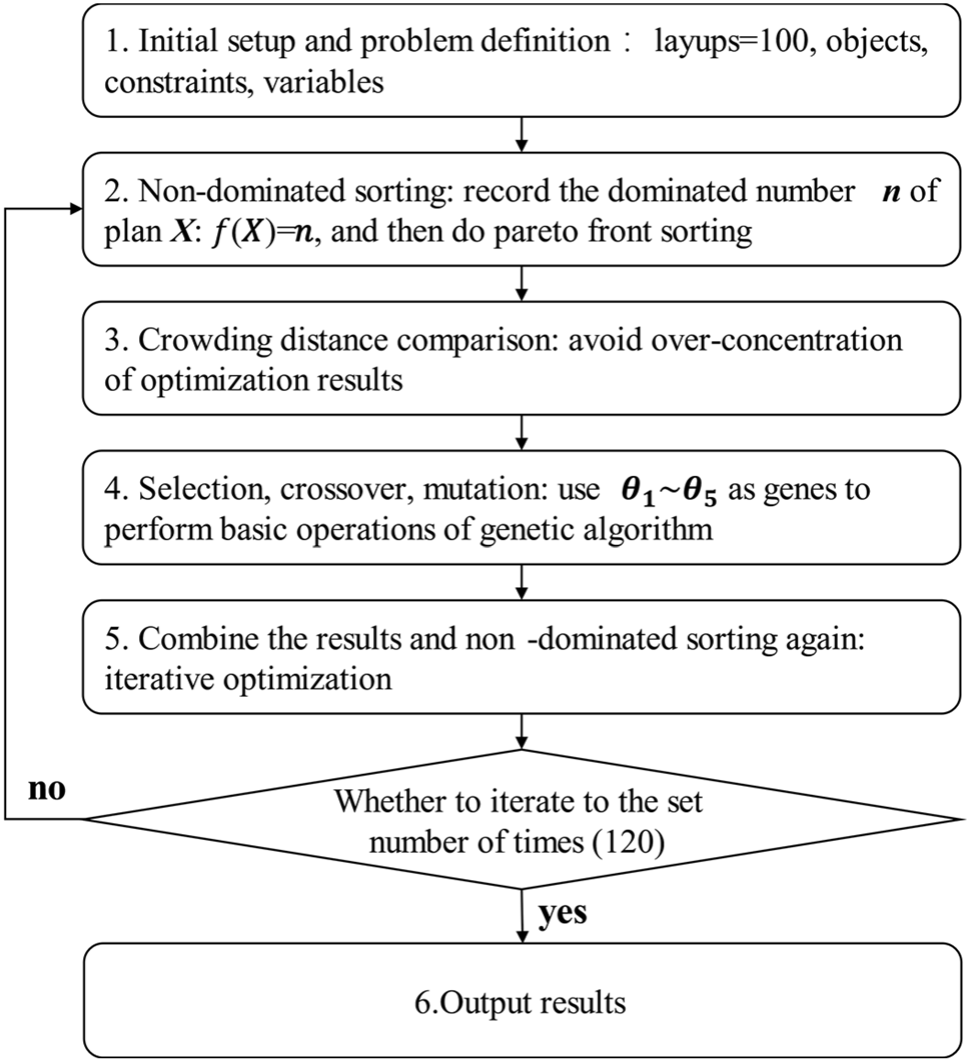
The application of NSGA-II algorithm in CFRP layup optimization.

This paper presents a program for optimizing CFRP layup using Python. The program consists of 6 optimization steps, as shown in Fig. 7.

1. Initial setup and problem definition: The initial population for the optimization scheme is composed of 100 randomly generated layup design schemes, each referred to as an individual. These individuals contain five optimization variables, referred to as genes, which are ply orientations $({\theta }_{i}, i=\text{1,2},\text{3,4},5)$. The subsequent selection, crossover, and mutation processes are carried out using these genes. The termination criterion for the process is set to 120 iterations to achieve the Pareto front.
2. Nondominated sorting categorizes the population into different non-dominated layers based on the dominance relationship between individuals. For example, if individual P has smaller values of both ${\alpha }_{x}$ and ${\alpha }_{y}$ compared to individual Q, then Q is considered to be dominated by P. The number of individuals, including P, that can dominate Q within the population is tallied, denoted as $f(Q)=n$. In short, genetic prioritization between individuals is determined by comparing their performance across individuals. In this study, the performance manifestation is the CTE ${\alpha }_{x}$ and ${\alpha }_{y}$ of the different layup schemes.
  As shown in Fig. 8a: For individual A, there are no other individuals whose ${\alpha }_{x}$ and ${\alpha }_{y}$ are both lower than A's, meaning there is no individuals that can dominate A, resulting in $f(A)=0$. Conversely, individual F is dominated by 2 individuals, B and C, whose ${\alpha }_{x}$ and ${\alpha }_{y}$ are both lower, denoted as $f(F)=2$. However, observing the layup scheme F, compared to layup schemes A and D, only one of ${\alpha }_{x}$ or ${\alpha }_{y}$ performs better, neither of which has a dominance relation. Similar assessments apply to the rest of the population.

**Figure 8:**
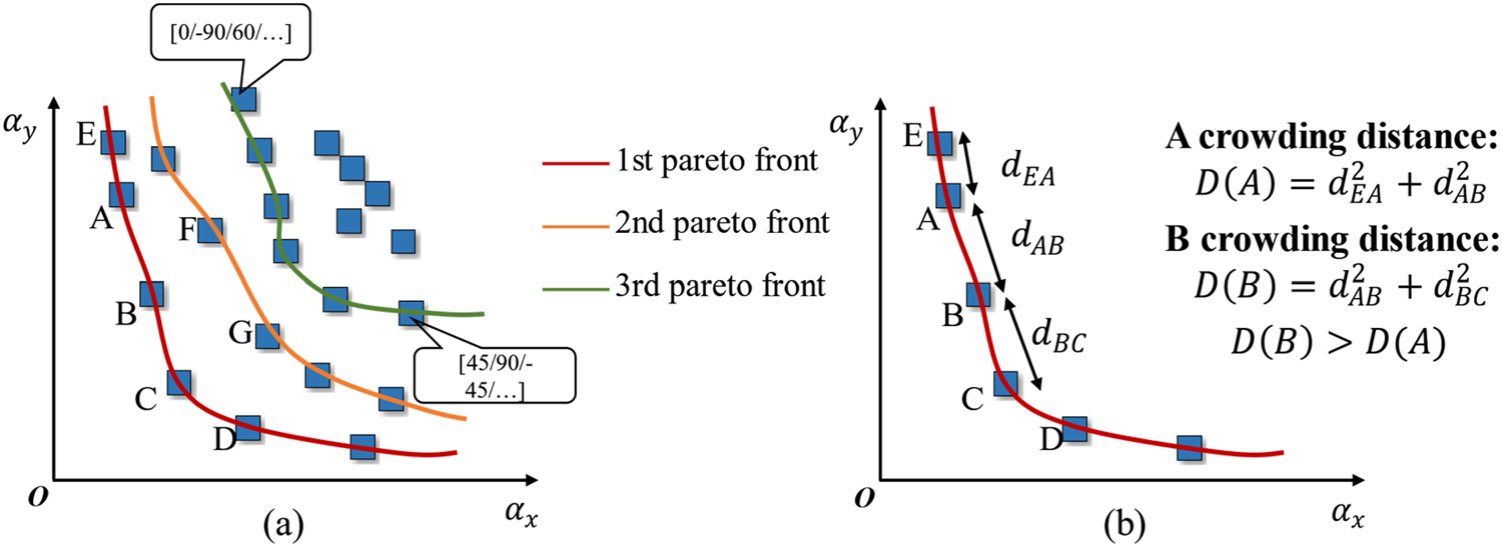
Non-dominated sorting (**a**) and crowding distance (**b**) schematic.
  Individuals with the same dominance counts are grouped into equivalent sets and ordered by their dominance count. These sets are labeled sequentially as the 1st Pareto front, 2nd Pareto front, and so forth, until all non-dominated Pareto fronts within the population are delineated. This procedure identifies the most preferable layup designs, positioning those within the earliest Pareto fronts as high-priority design options, earmarked for preferential consideration in subsequent genetic operations.
3. Crowding distance comparison: If individuals have the same non-domination rank, then their crowding distance D is compared, which is a measure that indicates the density of individuals around them. For research convenience, this paper calculates the crowding distance using the sum of squares method. As shown in Fig. 8b, if individuals A and B both reside on the 1st Pareto front, the crowding distances are calculated as follows:
  15 $$ \begin{array}{*{20}c} {D\left( A \right) = \left( {\alpha_{x}^{A} - \alpha_{x}^{E} } \right)^{2} + \left( {\alpha_{x}^{A} - \alpha_{x}^{B} } \right)^{2} + \left( {\alpha_{y}^{A} - \alpha_{y}^{E} } \right)^{2} + \left( {\alpha_{y}^{A} - \alpha_{y}^{B} } \right)^{2} } \\ \end{array} $$
  16 $$ \begin{array}{*{20}c} {D\left( B \right) = \left( {\alpha_{x}^{B} - \alpha_{x}^{A} } \right)^{2} + \left( {\alpha_{x}^{B} - \alpha_{x}^{C} } \right)^{2} + \left( {\alpha_{y}^{B} - \alpha_{y}^{A} } \right)^{2} + \left( {\alpha_{y}^{B} - \alpha_{y}^{C} } \right)^{2} } \\ \end{array} $$
  As illustrated in the figure, D(B) > D(A), indicating that although the layup schemes A and B have the same non-dominance ranking and are both on the 1st Pareto front, the priority of layup B is higher than that of A. This ensures a relatively uniform distribution of results within a certain range.
4. Selection, crossover, and mutation operations of genes: Layup design schemes with high comprehensive priority are selected through the non-dominated sorting and crowding distance comparison. Subsequently, crossover and mutation operations are performed on their five ply orientations (θ).
5. Merging the population and optimizing iteratively: After performing selection, crossover, and mutation, new layup designs are obtained. These new and existing layup schemes are then merged, and non-dominated sorting and crowding distance calculations are performed again. This process is iterated until the optimal Pareto front is reached, as shown in Fig. 9.

**Figure 9:**
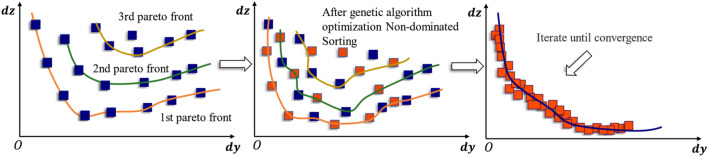
Iterative optimization to the optimal Pareto front.
6. The obtained output results yield several layup designs, all of which are relatively optimal. Only those layups that meet the requirements for ${\alpha }_{x}$ and ${\alpha }_{y}$ should be incorporated into the structural design.

## Experiment and result

Considering that each ply orientation may deviate from the design value in practical applications, potentially affecting material performance, it is necessary to conduct validation experiments. These experiments should analyze errors in layup angles in conjunction with actual manufacturing processes. This approach will facilitate a more in-depth discussion of the applicability of the CFRP optimization method in ultra-stable structure applications.

### Optimization design results

Select the layup from the optimization results conducted using the NSGA-II algorithm, as shown in Table 2. The first four layups are conventional angle layups, which struggle to meet all three design objectives simultaneously due to the angle constraints. Layups 5–8 feature unconventional layups that are not constrained by angles during the optimization process, thus allowing for a broad range of feasible design results that meet the requirements. The last two layups, designed to demonstrate the superiority of the algorithm, achieve extremely low axial CTE, illustrating the method's effectiveness in optimizing CFRP. However, due to their excessive lateral thermal expansion, these layups are not practical for structural applications and are therefore not discussed in detail.

**Table 2:** Different layups and CTE performance results.

Layup	Ply staking sequence	${\alpha }_{x} ({10}^{-6}/\text{K})$	${\alpha }_{y} ({10}^{-6}/\text{K})$
1	${\text{[45/}-\text{45/0/90/90/0/}-\text{45/45/90/0]}}_{s}$	1.439	1.439
2	${\text{[45/}-\text{45/0/90/0/60/}-\text{60/0/90/0]}}_{s}$	1.103	1.843
3	${\text{[45/}-\text{45/0/60/}-\text{60/0/90/}-\text{45/45/0]}}_{s}$	0.9777	1.993
4	${\text{[45/}-\text{45/30/}-\text{30/60/}-\text{60/0/45/-45/0]}}_{s}$	0.1972	3.231
5	${\text{[37.08/}-\text{59.45/31.38/}-\text{57.01/16.64/}-\text{16.64/57.01/}-\text{31.38/59.45/}-\text{37.08]}}_{s}$	0.5314	2.601
6	${\text{[32.61/}-\text{53.67/37.04/}-\text{56.86/32.65/}-\text{32.65/56.86/}-\text{37.04/53.67/}-\text{32.61]}}_{s}$	0.5242	2.502
7	${\text{[30.49/}-\text{51.63/36.10/}-\text{60.38/30.44/}-\text{30.44/60.38/}-\text{36.10/51.63/}-\text{30.49]}}_{s}$	0.4338	2.662
8	${\text{[40.56/}-\text{37.26/50.84/}-\text{25.04/55.81/}-\text{55.81/25.04/}-\text{50.84/37.26/}-\text{40.56]}}_{s}$	0.3548	2.736
9	${\text{[45.40/}-\text{7.540/}-\text{64.58/26.59/}-\text{26.14/64.58/}-\text{26.59/7.540/26.14/}-\text{45.40]}}_{s}$	0.0076	3.970
10	${\text{[13.40/}-\text{10.49/13.98/}-\text{65.31/1.055/}-\text{13.40/10.49/}-\text{13.98/65.31/}-\text{1.055]}}_{s}$	0.0032	6.443

Layup 8 has been identified as meeting the specified requirements for both axial and radial thermal expansions more effectively. This design ensures that the axial CTE is less than $0.6\times {10}^{-6}/K$ and maintains a lower radial CTE of ${\alpha }_{y}^{S}=2.736\times {10}^{-6}/K$, which facilitates a better thermal stress match with Invar joints.

When selecting layup 8 for the laminates used in the side support rods, to meet the requirement of ${\alpha }_{x}^{S}:{\alpha }_{x}^{P}=1:1.217$, the CTE of the main support rod should be ${\alpha }_{x}^{P}=0.4322\times {10}^{-6}/K$. The results of the second stage optimization specify layup 7.

### Experimental results and error analysis

Given that angle errors in lamination can affect material properties, the actual structural performance might deviate from the intended design outcomes. To ensure the optimized layups perform adequately in applications, sample tests were conducted on the first eight layups to evaluate their CTE.

**Figure 10:**
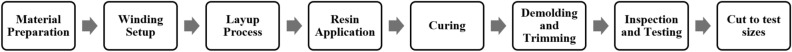
CFRP Processing and Specimen Cutting Procedure.

Considering the lack of precision in roll-wrapped CFRP prepreg for unconventional layups due to non-integer layup angles, choosing the winding process for manufacturing is more appropriate. This winding process, in which the angle of layup is controlled by controlling the rolling and traveling speed of the equipment, not only reduces errors, but also ensures uniform material properties, which is particularly effective for processing CFRP pipe fittings. The CFRP tubes were then cured, demolded, and post-treated, and their ends were cut to standard dimensions based on the method in the references^21^ to obtain the CTE test specimens. The test sample production flow is shown in Fig. 10.

**Figure 11:**
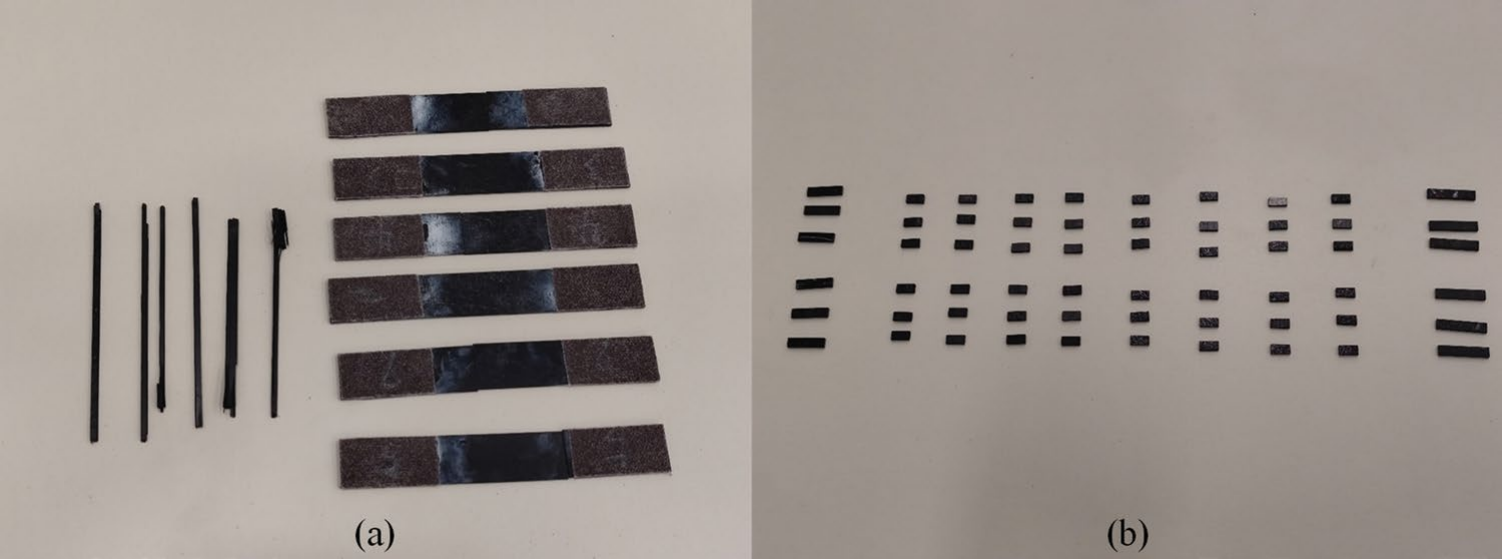
Layup samples for testing mechanical properties of lamina (**a**) and CTE tests for laminates (**b**).

As can be seen from Eq. (14) in the sectoin "Mathematical modeling of thermal deformation of CFRP", it is important to obtain basic lamina mechanical and thermal expansion data before proceeding with the layup design. Therefore, mechanical property parameter tests and CTE tests were conducted for the selected M40J/C1414 material. The Fig. 11a is the sample of tensile test of lamina material based on the standard GB/T 3354–2014. The Fig. 11b is the CTE test of lamina material and 8 types of layups based on the testing standard GB/T16535-2008. Considering the anisotropic characteristics of CFRP, all of the above include test results in both longitudinal and transverse directions.

The CTE of specimens from three different batches was measured through experiments using the non-contact LINSEIS L75 Laser Dilatometer. Each sample underwent three measurement cycles at temperature conditions ranging from 20 to 80 °C. The average CTE values, calculated from these three cycles, are presented in Tables 3 and 4.

**Table 3:** Experimental results of CFRP samples of ${\alpha }_{x}$.

${\alpha }_{x}$($1\times {10}^{-6}/K$)	Layup1	Layup2	Layup3	Layup4	Layup5	Layup6	Layup7	Layup8
1st batch	1.36	0.835	1.210	0.230	0.631	0.957	0.506	0.241
2nd batch	1.62	0.980	0.856	0.456	0.984	1.080	0.828	0.231
3rd batch	1.85	0.957	1.221	0.126	0.977	1.370	0.982	0.343
Average	1.61	0.924	1.096	0.271	0.864	1.136	0.772	0.272
Theoretical results	1.44	1.103	0.977	0.197	0.531	0.524	0.434	0.355

**Table 4:** Experimental results of CFRP samples of ${\alpha }_{y}$.

${\alpha }_{y}$($1\times {10}^{-6}/K$)	Layup1	Layup2	Layup3	Layup4	Layup5	Layup6	Layup7	Layup8
1st batch	1.47	2.88	2.14	3.04	3.81	3.78	3.49	3.69
2nd batch	1.87	2.14	2.54	3.18	3.71	4.05	4.26	3.06
3rd batch	2.42	2.03	2.12	3.56	3.28	3.05	2.88	3.28
Average	1.92	2.35	2.27	3.26	3.60	3.63	3.54	3.34
Theoretical results	1.44	1.84	2.00	3.23	2.60	2.50	2.66	2.74

**Figure 12:**
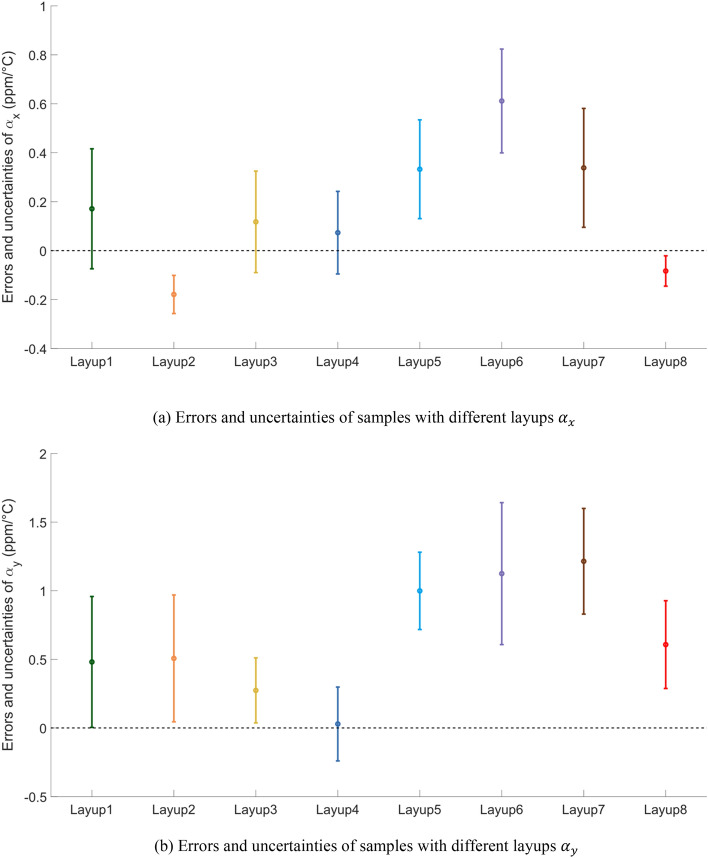
Error and Uncertainty Analysis of CTE of Samples in ${\alpha }_{x}$ and ${\alpha }_{y}$.

Here is the error and uncertainty analysis of 8 types of layups, as shown in Fig. 12.

The test results of CFRP specimens with common ply orientations show more stability, indicating that the current level of lamination technology for conventional angles such as ± 45°, ± 60°, ± 30°, 0°, and 90° is superior to that of unconventional angle layup designs. In terms of thermal expansion performance, the CTE is smaller for specially designed unconventional layups. However, these unconventional layups schemes are more challenging to achieve when considering manufacturing errors.

### Application in gravitational wave telescope design

**Figure 13:**
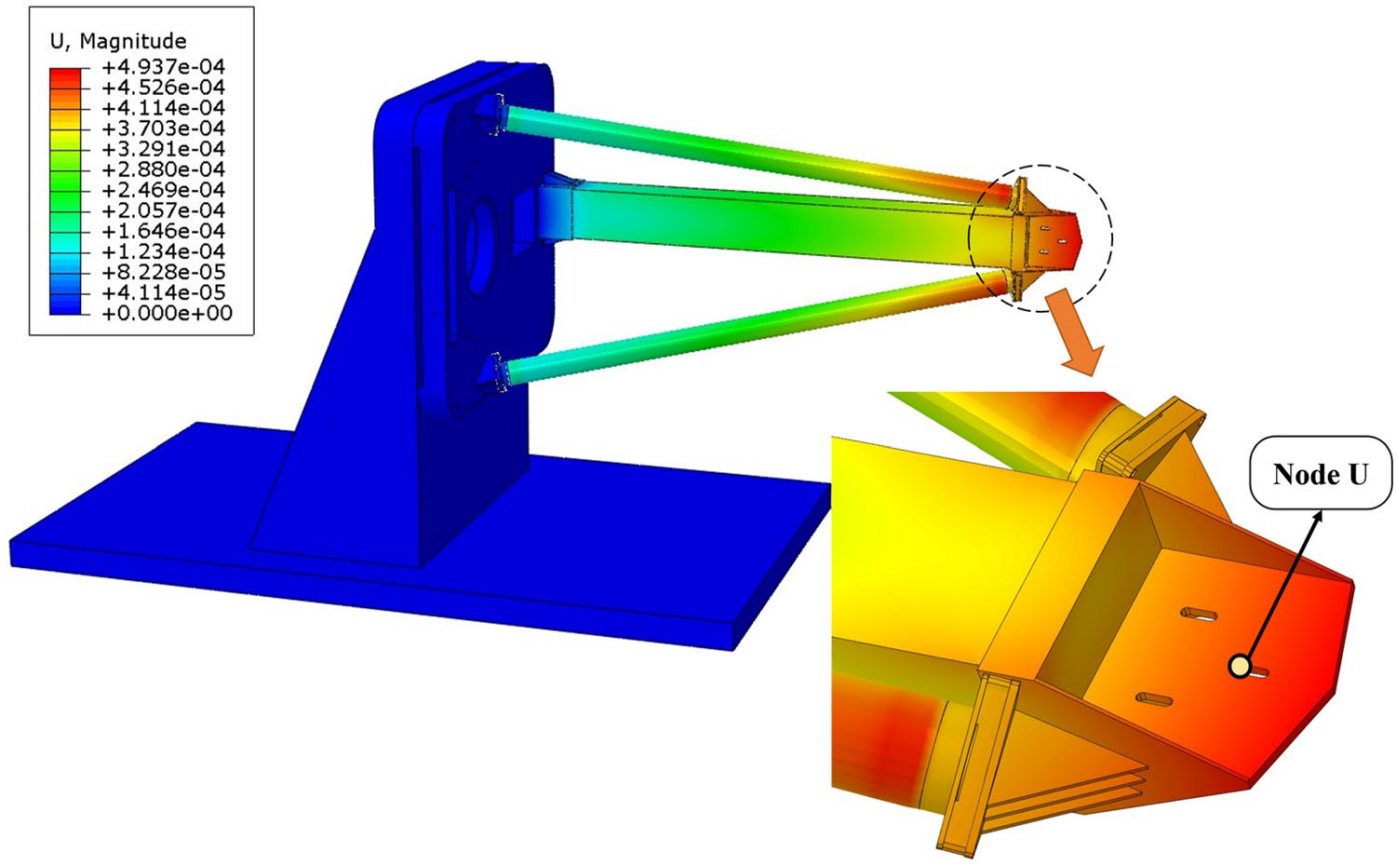
Structural thermal deformation after bringing experimental results into finite element simulation.

Before conducting thermal deformation analysis of the structure, data of CFRP from 1st batch of layups 7 and 3rd batch of layup 8 was integrated into the structure. As demonstrated in Fig. 13, a comprehensive finite element simulation was conducted using ABAQUS. The general performance results are presented in Table 5.

**Table 5:** Summary of structural mechanical properties.

Specification	Requirement	Result
1. Mass with 3 support rods	< 7.5 kg	6.37 kg
2. First Eigen frequency	> 80 Hz	141.66 Hz
3. Deformation due to gravity	$\Delta z_{G} < 10\;\mu {\text{m}}$ $\Delta y_{G} < 10\;\mu {\text{m}}$	$\Delta z_{G} = 0.31\;\mu {\text{m}}$ $\Delta y_{G} = 8.81\;\mu {\text{m}}$
4. Strength requirements	${\upsigma }_{{{\text{Max}}}} {\text{ < 35MPa}}$	4.96 MPa

Solid element (C3D8R) meshes were used for the entire structure, excluding the three CFRP support rods. The support rods are modeled as an anisotropic material shell element (S4R), with the basic mechanical properties of the material consistent with theoretical values, and the CTE chosen based on actual test results. The material definition for the CFRP support rods is presented in Table 6.

**Table 6:** Material definition for the support rods.

	${E}_{x}$(GPa)	${E}_{y}$(GPa)	${G}_{xy}$	${\alpha }_{x}$(ppm/K)	${\alpha }_{y}$(ppm/K)
Main support rod	44.01	32.07	40.19	0.506	3.49
Side support rods	38.41	28.51	41.90	0.343	3.28

In order to accurately obtain the deformation of the truss, the structure support metal frame was constrained to prevent any participation in the deformation. Thermal deformation was evaluated based on the displacement data at Node U, located at the secondary mirror’s position, as detailed in Table 7.

**Table 7:** Results of structure thermal deformation for different cases.

	Structure type (${10}^{-6}/K$)	dy/K (nm)	dz/K (nm)
1	All-Invar structure (${\alpha }_{Invar}=0.6$)	199.4	528.8
2	Unoptimized truss structure with layup1	423.2	1198.4
3	Structure simulation result with layup optimization	4.229	454.7
4	Truss theoretical calculation results	3.416	363.5
5	Structure simulation with actual specimen data	70.39	487.7

Due to the high specific stiffness of the CFRP material and the overall light weighting of the structure, the three items of structural weight, modal and deformation under gravity are satisfied with the design requirements.

It should be noted here that the maximum stress indicator presented in row 4 is based on the strength requirements of the M40J/C1414 lamina material. Given that the reinforcement of fibres in the transverse direction is relatively modest, the strength of CFRP material is the lowest in that direction, with a value of 45 MPa. Given the strength margin, it is deemed appropriate to set the maximum strength design target at 35 MPa. A finite element simulation analysis was conducted to assess the working condition of the telescope structure prototype. The analysis revealed that when thermal stress is combined with the influence of gravity, the maximum stress of the structure as a whole is 4.96 MPa. Consequently, the current structural design is deemed to meet the maximum strength requirements.

It is clear that the performance of CFRP in actual applications will deviate from the design values, and such deviations introduced into the structure can have unexpected effects on the thermal deformation of the telescope. Therefore, it is necessary to incorporate the actual measurement results of CFRP into the telescope structure, compare them with the theoretical design results, and determine the acceptable range of errors.

Table 7 shows the optimization results for thermal deformation of the telescope structure. The material of the three rods was replaced to compare different design schemes, while keeping all other components unchanged.

The optimized structure achieved the best performance under ideal conditions, exhibiting the lowest thermal deformation compared to all other scenarios. It reduced dz by 14% and dy by 98% compared to the all-Invar structure. Furthermore, an examination of rows 2 and 3 reveals that the optimization of thermal deformation in the structure through the use of CFRP, with a CTE ratio of 1.217:1 for the main support rods to the side support rods, is evident. This is in contrast to the unoptimized quasi-isotropic layup, where dz was reduced by 62.05% and dy by 99%. Observing rows 3 and 5, introducing materials with error into the structural simulation will indeed affect the thermal deformation performance and make the structure fail to achieve the desired thermal deformation results. However, in comparison, the optimization effect is still significant and the structure still meets the design requirements.

### Discussion of limitations of optimization methods

Following the analysis presented, it is apparent that deviations in the CFRP layup have a substantial impact on the material's actual performance. Therefore, an in-depth analysis should be conducted in conjunction with the error numerical analysis method to identify the limitations of the optimization method proposed in this paper.

**Figure 14:**
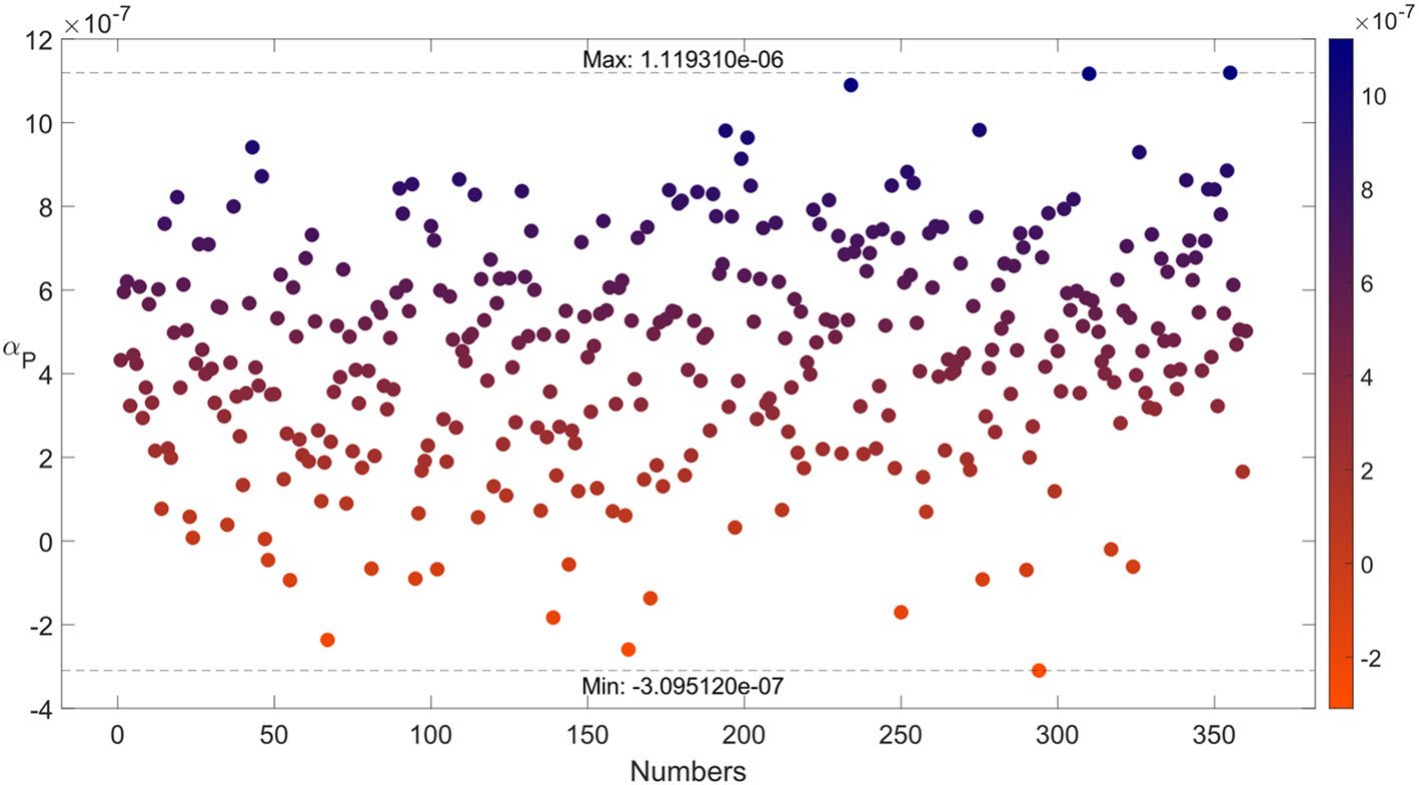
Error analysis based on DOE: Variation within ± 5°.

Given the significant impact of the axial CTE of the main support rod on the structure's thermal deformation, the discussion will henceforth focus solely on ${\alpha }_{x}^{P}$, hereinafter referred to as ${\alpha }_{P}$, to control variables. Using the Design of Experiments (DOE) module within Isight, these stochastic errors were integrated into the previously determined layup of the main support rod, providing 360 random error calculations and statistically determining the possible outcome range for ${\alpha }_{P}$.The examination of the implications of a 5° lamination error is illustrated in Fig. 14, with similar analyses applicable to other error scenarios.

When the error is within ± 5°, the resulting ${\alpha }_{P}({10}^{-6}/K)$ interval is [-0.31, 1.12]. The theoretical prediction for ${\alpha }_{P}$ is 0.434 as layup 7 in Table 3, and the experimental test results for three different batches of samples are 0.83, 0.98, and 0.51, all within this range. This indicates that the experimental results align with the error prediction, albeit with significant dispersion.

**Figure 15:**
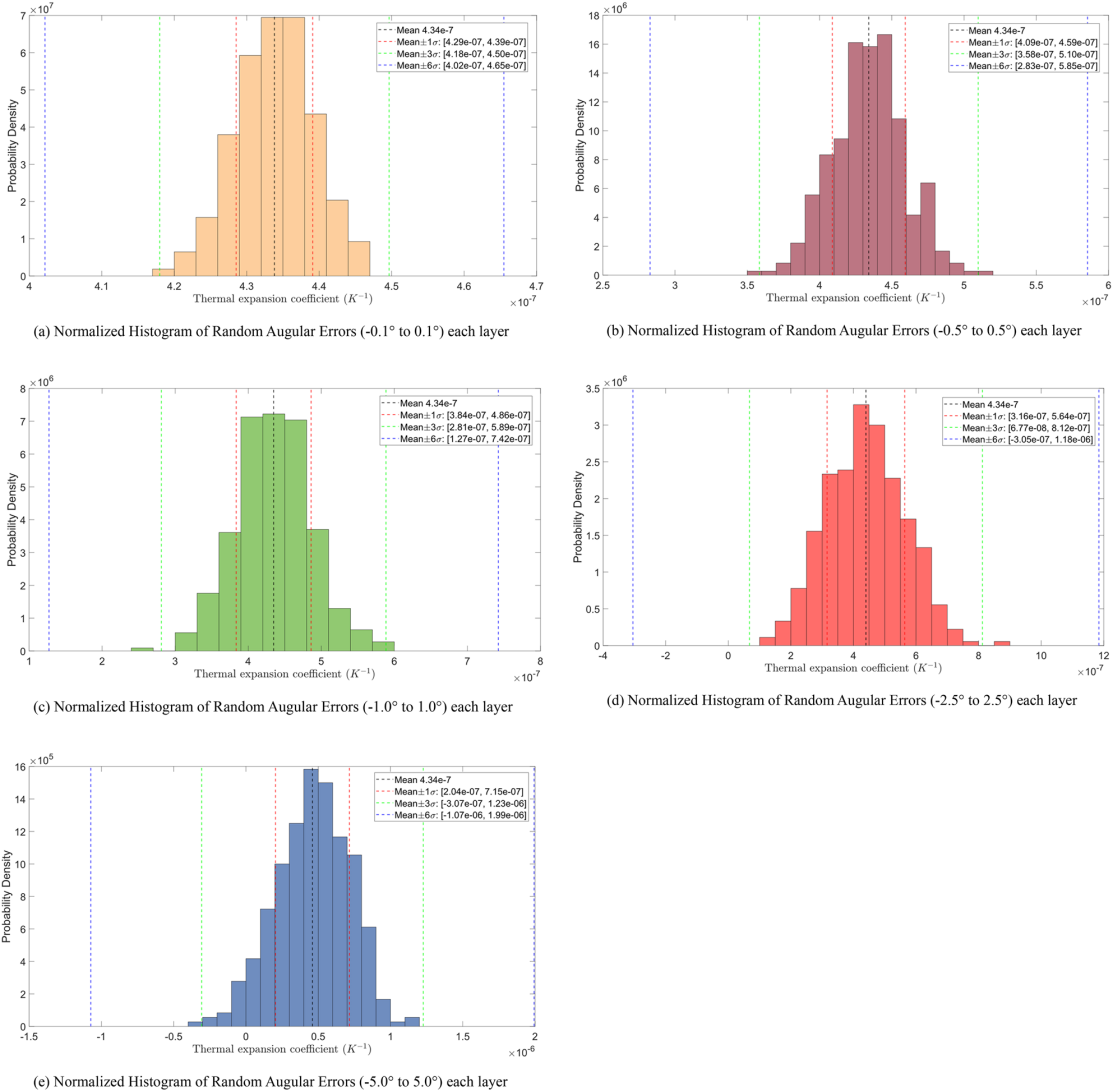
Histograms under different random ply error conditions.

To further investigate the applicability of the optimization method presented in this study, the results of the DOE under the conditions of each ply orientation error of ± 2.5°, ± 1°, ± 0.5°, and ± 0.1° are presented, as shown in Fig. 15. The higher 3σ boundary values from the DOE numerical experiments are incorporated into the truss model to analyze and obtain dy values for different error conditions, as shown in Table 8.

**Table 8:** Results of structure thermal deformation (ΔT = 1 K) under different layup error conditions.

Random error interval with per layer	3σ-$\alpha_{P}$(10^−6^/K)	dy (nm)	Dz (nm)
$[ - 5^{^\circ } , + 5^{^\circ } ]$	1.230	1718.52	1030.73
${[} - {2}{\text{.5}}^{^\circ } {, + 2}{\text{.5}}^{^\circ } {]}$	0.812	818.11	680.46
$[ - 1^{^\circ } , + 1^{^\circ } ]$	0.589	337.74	493.58
$[ - 0.5^{^\circ } , + 0.5^{^\circ } ]$	0.510	167.56	427.38
$[ - 0.1^{^\circ } , + 0.1^{^\circ } ]$	0.450	38.31	377.10
No error	0.434	3.416	363.52

The results show that:

1. When the layup error level is controlled within 0.1°, the optimization design method proposed in this paper significantly reduces structural thermal deformation. Although a substantial difference remains compared to the ideal state, the method sufficiently meets the design requirements of dy < 200 nm/K and dz < 550 nm/K, indicating its effectiveness.
2. The optimization method is no longer applicable when the error level exceeds 0.5° or above.
3. When the error exceeds 1°, there is a likelihood that the structural performance will not meet the design requirements.

It is clear that utilizing layup optimization to adjust ${\alpha }_{P}\ne {\alpha }_{S}$ for minimizing structural thermal deformation demands stringent precision in the CTE of the rods. It is worth noting that even a slight deviation of 0.1° in each layer can result in actual outcomes that deviate significantly from the ideal design solution, by an entire order of magnitude. Currently, based on actual production experience and the results of tests carried out by the LISA team on CFRP in the literature^5^, the typical processing error for unconventional layups is approximately 0.5°. Therefore, to utilize the structural optimization method proposed in this paper to reduce the thermal deformation of the truss structure, the level of lamination technology should be considered, and a higher precision lamination process should be employed in the production of the CFRP truss.

## Conclusion

This paper presents three design objectives for the CTE of CFRP, derived from the design requirements of the gravitational wave telescope truss structure. The subsequent sections describe the optimized design method, experimental verification, application in structure, and numerical error analysis. The following conclusions are drawn:

1. This study introduces a novel design method to address the thermal deformation challenges in the structure of gravitational wave detection telescopes, leveraging the properties of CFRP materials and truss structures. Initially, a mathematical model for the thermal deformation of a three-rod truss structure is established. The method differentiates the CTE between the main and side support rods, using the structural characteristics to mitigate thermal deformation in the eccentric direction of the telescope. Subsequently, considering the structural requirement for a specific ratio of CTE values, the NSGA-II algorithm is employed alongside the design characteristics of CFRP laminates to optimize the axial and radial CTEs, yielding a series of CFRP laminations that meet the design requirements. Afterwards, the design results are divided into conventional and unconventional laminations for sample testing and error analysis, and the most suitable laminations are selected for structural design. The findings confirm that the optimization method proposed in this paper is feasible under certain process conditions and the optimization effect is obvious.
2. It is recommended that unconventional angle laminates be employed to address the issue of the material's requirement for a fixed value of CTE. Comparing the test results of conventional layups and unconventional layups, it can be seen that although the processing error of unconventional layups is larger than conventional layups, the thermal expansion performance of the unconventional layups can meet multiple design goals simultaneously. Therefore, the unconventional layups have great development potential for structures that do not require high mechanical efficiency.
3. In terms of structural applications, the optimized CFRP design can be used to meet the different CTE requirements of the main and side support rods in the structure, which can significantly reduce the thermal deformation of the structure. This optimization not only enhances the dimensional stability and operational reliability of gravitational wave detection telescopes but also serves as a valuable case study for enhancing similar structures in aerospace engineering. This is particularly relevant for telescope structures that demand stringent thermal deformation control.
4. Considering the large processing error of unconventional laminates in actual situations, this causes the actual CTE of laminates to deviate from the design value, thereby affecting the structural performance. Within the limitations of the study, this paper uses the DOE module in Isight to perform a numerical simulation of the laminate error on the axial thermal expansion coefficient of the selected laminate, with the objective of determining the applicable scope of the method proposed in this paper.
5. This paper has some shortcomings and limitations in its research content. It primarily focuses on principle prototype tests of the gravitational wave detection telescope, with experimental objectives centered on detecting thermal deformation of the structure. Consequently, discussions from material optimization to structural design concentrate primarily on thermal deformation, paying less attention to strength, stiffness, light weighting, and other mechanical properties. Additionally, discussions about errors in CFRP layup angles have relied extensively on numerical simulations, as outlined in section “Discussion of limitations of optimization methods”, without experimental validation. Future research could benefit from integrating tools like metallographic microscopes and employing techniques such as machine learning and visual imaging to detect errors in CFRP layup angles, thereby enhancing the performance stability of carbon fiber products.

## Data Availability

The datasets used and/or analyzed during the current study available from the corresponding author on reasonable request.
